# Histopathological characterization of intimal lesions and arterial wall calcification in the arteries of the leg of elderly cadavers

**DOI:** 10.1002/ca.23701

**Published:** 2020-11-20

**Authors:** Annelotte Vos, Pim A. de Jong, Daphne Verdoorn, Willem P. T. M. Mali, Ronald L. A. W. Bleys, Aryan Vink

**Affiliations:** ^1^ Department of Pathology University Medical Center Utrecht Utrecht The Netherlands; ^2^ Department of Radiology University Medical Center Utrecht The Netherlands; ^3^ Department of Anatomy University Medical Center Utrecht The Netherlands

**Keywords:** atherosclerosis, histology, lower extremity, medial arterial calcification

## Abstract

**Introduction:**

Although arteries of the leg have been studied in extensively diseased amputation specimens, little is known about the composition of vascular lesions present in the general population. The aim of this study was to describe the natural development of adaptive intimal thickening, atherosclerotic lesion development and vascular calcification in the leg of a general elderly population.

**Materials and Methods:**

Two hundred and seventy postmortem samples from the popliteal and posterior tibial arteries of 14 elderly cadavers were studied histologically.

**Results:**

Atherosclerotic lesions were more frequently observed in the popliteal (60%) than in the posterior tibial artery (34%; *p* < .0005). These atherosclerotic plaques were most often nonatheromatous (80% and 83% for popliteal and posterior tibial plaques, respectively). The atheroma's that were present were small (most <25% of plaque area). Atherosclerotic plaque calcification was observed more often in the popliteal (39%) than in the posterior tibial samples (17%; *p* < .0005). Medial arterial calcification was observed more often in the posterior tibial (62%) than in the popliteal samples (46%; *p* = .008). Plaque calcification and medial arterial calcification were not associated with lumen stenosis.

**Conclusions:**

In the leg of elderly cadavers, the presence of atherosclerotic plaque and intimal calcification decreases from the proximal popliteal artery to the more distal posterior tibial artery and most atherosclerotic lesions are of the fibrous nonatheromatous type. In contrast, the presence and severity of medial calcification increases from proximal to distal.

## 1 INTRODUCTION

With advancing age arteries undergo modifications. Adaptive intimal thickening is an adaptation of the arterial wall to mechanical stresses that are induced by variations in flow and wall tension (Stary et al., 1992). Thickening of the intima can be observed in the arteries of healthy human subjects and from many other species and could therefore be considered as a physiological adaptation. In contrast to intimal thickening, atherosclerosis is considered a pathological process which might eventually lead to occlusion of the arterial lumen. Atherosclerotic lesions can be divided in fibrous lesions, consisting of connective tissue and smooth muscle cells, and atheromatous lesions with a large pool of extracellular lipids and presence of macrophages (Virmani, Kolodgie, Burke, Farb, & Schwartz, 2000). Calcifications can also be present in atherosclerotic plaques, predominantly in plaques of the fibrous subtype. In the arterial wall calcifications are not only observed in atherosclerotic plaque, but also in the medial layer. These calcifications result in decreased vessel compliance (Leopold, 2015).

Previous studies have described the presence of subclinical atherosclerotic lesions on radiological imaging in the lower extremity of a general elderly population (Han et al., 2018). Detailed histological information about the natural development and distribution of arterial lesions in the leg of the general population is lacking. In a previous histological study we have described the natural development of intimal lesions in the arterial system of non‐diseased elderly individuals, but in this previous study only the femoral artery was included and not the below the knee arteries (Vink et al., 2002). Some older studies from the 1960s have studied below the knee arteries of asymptomatic individuals, however these studies mainly focused on the medial wall layer (Meyer & Stelzig, 1967; Wright, 1963). The aim of the present study was to describe the distribution of adaptive intimal thickening, atherosclerosis and atherosclerotic‐ and medial calcifications in the leg in a non‐selected general population. Arterial modification develops slowly during life. Therefore, we used elderly cadavers.

## 2 MATERIALS AND METHODS

### 2.1 Patient population

Twenty‐four leg specimens were obtained from 14 cadavers aged 70–96 years at death (median 87 years). 6/24 (25%) leg specimens were from male donors. The history of cardiovascular disease and risk factors was unknown.

### 2.2 Tissue collection and processing

Postmortem popliteal and tibial artery specimens were obtained from 14 elderly cadavers, who gave written informed consent regarding the use of their bodies for educational and research purposes. After pressure formaldehyde (4%) fixation of the bodies, a total of 24 popliteal and 24 tibial arteries were collected (bilateral in 10 cadavers). The arteries were decalcified using diaminoethylene tetraacetic acid solution (EDTA) for at least 48 hr. Decalcification was necessary to maintain the morphology and interpretability of the arterial wall layers. Since histologic evaluation of calcification is based on visualization of matrix previously altered by the calcification process, and not calcium ions itself, decalcification does not influence analysis (Burke, Taylor, Farb, Malcom, & Virmani, 2000; Meershoek et al., 2016). Two randomly selected segments were obtained from each artery; 1 cm segments of both the posterior tibial artery and popliteal artery were collected 8 and 13 cm distal and proximal of the origin of the anterior tibial artery, respectively. The harvested segments were divided into three samples of approximately 3–4 mm, perpendicular to the lumen. Due to technical issues, adequate histological examination was not possible in all vascular samples. A total of 132 samples of the popliteal and 138 samples of the tibial artery were examined. In most cadavers the posterior tibial artery was examined. In four legs the anterior tibial artery was examined, because the posterior tibial artery was not available due to technical issues.

### 2.3 Histopathological analysis

Microscopic slides of the vascular samples were stained with hematoxylin and eosin (H&E), Elastic van Gieson (EvG) to visualize elastin, Sirius red for collagen and CD68 immunostain for macrophages. Digitalized slides (Scan‐Scope XT scanner, Aperio Technologies) were analyzed using Aperio Image Scope software as described previously (Huisman, Looijen, van den Brink, & van Diest, 2010). The EvG stained slides were used to measure the luminal circumference and the intimal and medial surface and circumference. The percentage stenosis was calculated by dividing the intimal surface by the area encompassed by the internal elastic lamina. The Sirius red stain was used to determine the area of atheroma.

On the H&E stained slides the surface of vascular calcification in the intima and media and around the internal elastic lamina was measured, as well as the percentage of circumference of the media that was calcified due to calcifications in media and internal elastic lamina as described previously (Vos et al., 2016). For further analyses both calcification in the media and around the internal elastic lamina were considered medial calcifications (Micheletti, Fishbein, Currier, & Fishbein, 2008). Calcifications were characterized by sharp demarcated acellular spots and areas, which were dark pink to purple colored on H&E stained slides. Only calcifications of at least 1% of the total surface (intima) or circumference (media) were included in the analysis.

Intimal lesions present on each cross section were classified according to the modified American Heart Association Classification system (Virmani et al., 2000). These intimal lesions were further grouped into nonatherosclerotic intimal lesions (intimal thickening and intimal xanthoma) and atherosclerotic lesions (pathological intimal thickening, fibrous cap atheroma, thin fibrous cap atheroma and fibrocalcific plaque).

### 2.4 Statistics

Continuous data are presented as median and interquartile range and categorical data are presented as a frequency and percentage, unless specified otherwise. Due to skewed distributions and relatively small study groups, nonparametric tests were used; Chi Square tests for associations between categorical variables and Wilcoxon signed rank test (paired data) or Kruskal–Wallis tests for differences in continuous outcomes. When comparing more than two groups, post‐hoc tests with Bonferoni correction were used. Statistical analyses were conducted using SPSS Statistics version 24.0 (IBM Corporation, New York). *p* values of <.05 were considered significant.

## 3 RESULTS

### 3.1 Atherosclerosis

**FIGURE 1:**
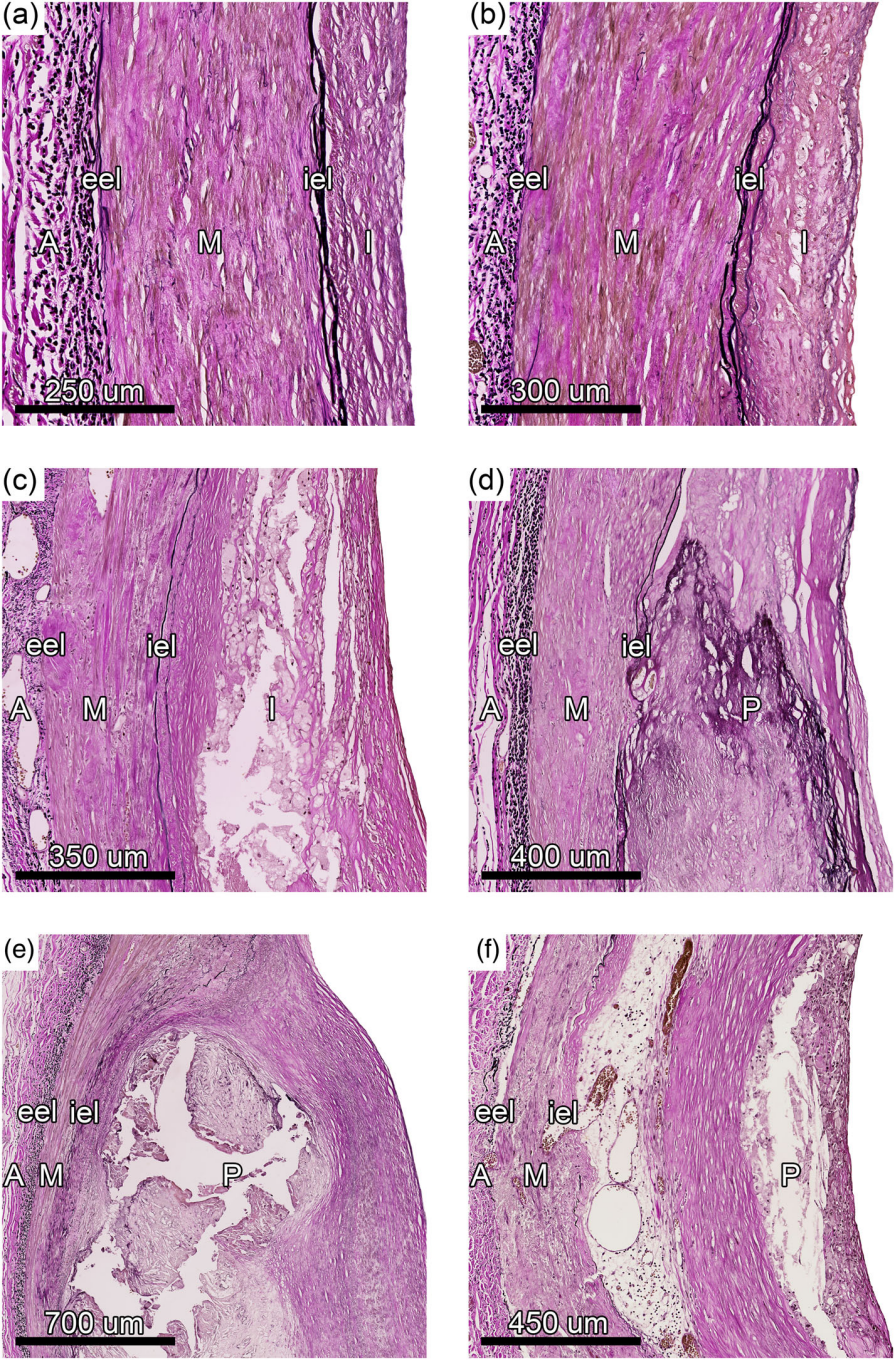
Atherosclerotic and nonatherosclerotic intimal lesions in the leg. Examples of the different intimal lesions found in the posterior tibial and popliteal artery. (a) Nonatherosclerotic intimal thickening, (b) nonatherosclerotic intimal xanthoma, (c) atherosclerotic pathological intimal thickening, (d) atherosclerotic fibrocalcific plaque, (e) atherosclerotic fibrous cap atheroma, (f) atherosclerotic thin fibrous cap atheroma. I = intima, P = plaque, iel = internal elastic lamina, M = media, eel = external elastic lamina, A = adventitia. Elastin van Gieson stain

In all samples, either nonatherosclerotic intimal thickening/xanthoma or atherosclerosis was observed. Atherosclerotic lesions were more often found in samples of the popliteal artery (79/132 (60%)) than in samples of the more distally located posterior tibial artery (47/138 [34%], *p* < .0005; Figure 1, Table 1). In both arteries these atherosclerotic lesions were most often non‐atheromatous lesions that were classified as pathological intimal thickening or fibrocalcified plaque (63/79 (80%) in the popliteal artery and 39/47 (83%) in the posterior tibial artery). A lipid core was present in 19/79 (24%) atherosclerotic samples of the popliteal and 8/47 (17%) atherosclerotic samples of the posterior tibial artery. These atheroma's were relatively small; in the popliteal artery the median percentage atheroma was 8% (4–19%) and in the posterior tibial artery 11% (3–23%) of the plaque surface. In 8/19 (42%) samples of the popliteal artery and in 4/8 (50%) samples of the posterior tibial artery with a lipid core, the percentage atheroma exceeded 10%. In only one case (a sample of the popliteal artery) the percentage atheroma exceeded 40%, a threshold historically correlated with an increased risk of ulceration and thrombosis (Davies et al., 1993). Strikingly, in one leg a ruptured fibrous cap atheroma with total occlusion and recanalization was observed in the posterior tibial artery.

**TABLE 1:** Arterial morphology and histology

		Popliteal artery	Posterior tibial artery^a^
Luminal diameter (mm)		4.8 (3.6–5.6)	1.9 (1.4–2.7)
Intima/media ratio		1.1 (0.5–2.3)	0.6 (0.3–1.4)
Percentage stenosis (%)		33 (19–51)	25 (15–60)
Atherosclerotic plaque		79/132 (60%)	47/138 (34%)
	Thin fibrous cap atheroma	1/132 (1%)	1/138 (1%)
	Fibrous cap atheroma	15/132 (11%)	7/138 (5%)
	Fibrocalcific plaque	46/132 (35%)	21/138 (15%)
	Pathological intimal thickening	17/132 (13%)	18/138 (13%)
Nonatherosclerotic intimal lesion		53/132 (40%)	91/138 (66%)
	Intimal xanthoma	10/132 (8%)	4/138 (3%)
	Intimal thickening	43/132 (33%)	86/138 (62%)
Intimal calcification present		52/132 (39%)	23/138 (17%)
Medial calcification present		60/132 (46%)	85/138 (62%)

*Note*: Categorical variables are presented as absolute frequencies and percentages, continuous variables are presented as median and interquartile range.

^a^ In four cases the anterior tibial artery was used.

### 3.2 Stenosis

**FIGURE 2:**
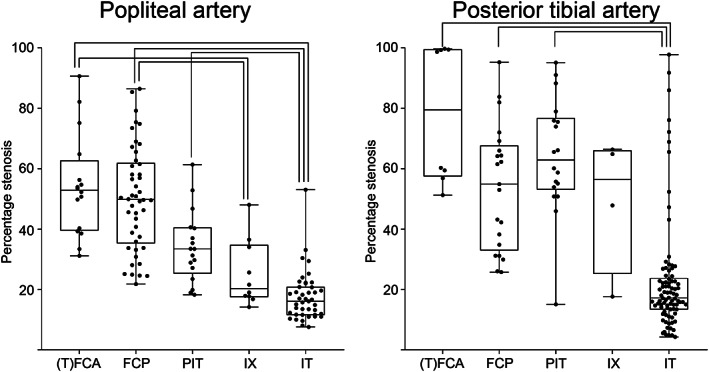
Percentage stenosis per atherosclerotic plaque type. The percentage stenosis was higher in samples with atherosclerotic plaque ((T)FCA = (thin) fibrous cap atheroma, FCP = fibrocalcific plaque, PIT = pathological intimal thickening) than in samples with intimal thickening for both arteries (IT; *p* < .0005). IX = intimal xanthoma. Significant differences are marked with a connecting line

The median percentage stenosis was 33% (19–51%) in the popliteal artery and 25% (15–60%) in the posterior tibial artery. Significant stenosis (>75% cross sectional luminal narrowing) was present in 7/132 (5%) samples of the popliteal artery (3 fibrous cap atheromas and 4 fibrocalcified plaques) and in 17/138 (12%) samples of the posterior tibial artery (4 fibrous cap atheromas, 3 fibrocalcified plaques, 6 pathological intimal thickening and 4 intimal thickening). The percentage stenosis was higher in samples with atherosclerotic plaques than in samples with intimal thickening (*p* < .0005; Figure 2).

### 3.3 Vascular wall calcification

**FIGURE 3:**
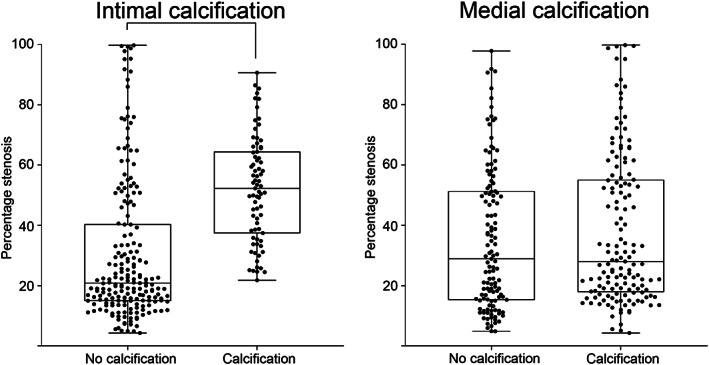
Percentage stenosis in relation to presence of vascular wall calcification. The percentage stenosis was significantly higher in samples with intimal calcification than in samples without intimal calcification (*p* < .0005). There was no relation between the presence of medial arterial calcification and the percentage stenosis (*p* = .256). Significant differences are marked with a connecting line

Intimal calcification was more frequently present in samples of the popliteal artery (52/132 (39%)) than in samples of the posterior tibial artery (23/138 (17%); *p* < .0005). As expected, there was a clear relation between intimal calcification and atherosclerosis, with intimal calcification in 75/126 (60%) of samples with atherosclerotic plaque and in none of the samples without atherosclerosis (*p* < .0005). The percentage of stenosis was higher in samples with intimal calcification than in samples without (*p* < .0005; Figure 3).

Medial arterial calcification was more often present in samples of the posterior tibial artery (85/138 (62%)) than in samples of the popliteal artery (60/132 [46%]; *p* = .008). In addition, in the posterior tibial artery a higher percentage of the medial circumference was calcified (34% (9–82%) vs. 11% (4–21%) in the popliteal artery; *p* < .0005). There was no relation between the occurrence of medial arterial calcification and atherosclerotic intimal lesion, with medial calcification in 55% of the samples with atherosclerotic plaque and in 53% without atherosclerosis (*p* = .744). There was no relation between the presence of medial arterial calcification and the percentage stenosis (*p* = .256; Figure 3).

**FIGURE 4:**
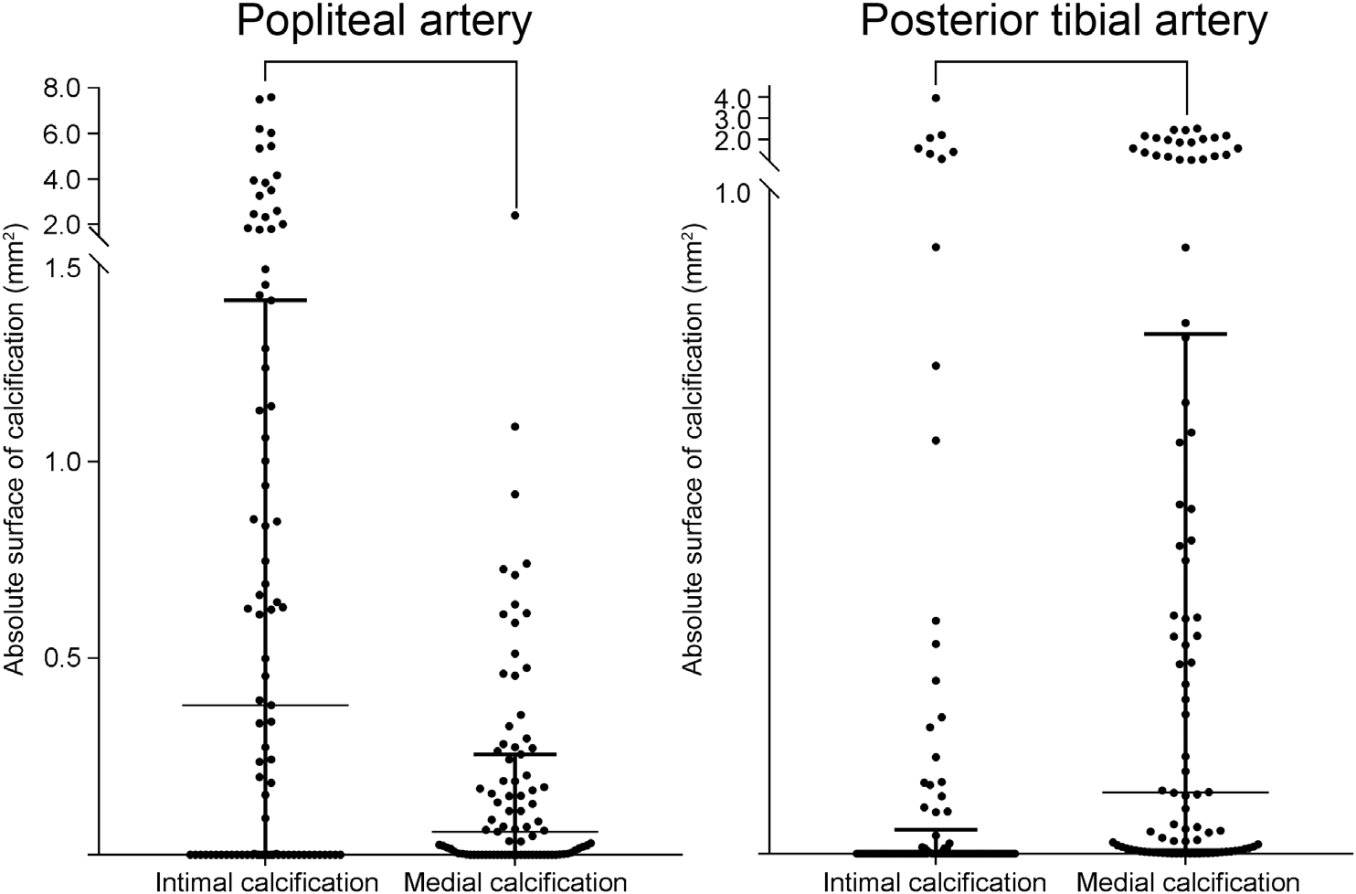
Absolute surface of calcification in the intima and media. In the popliteal artery the amount of intimal calcification was significantly higher than the amount of medial calcification (*p* < .0005), while in the posterior tibial artery the amount of medial arterial calcification was significantly higher than the amount of intimal calcification (*p* < .0005). Significant differences are marked with a connecting line

When comparing the absolute amounts of intimal and medial arterial calcification in samples with calcification in intima and/or media, in the popliteal artery the amount of calcification in the intima was significantly higher than in the medial layer of the vascular wall (*p* < .0005; Figure 4). In the posterior tibial artery, however, the amount of medial arterial calcification was significantly higher than the amount of intimal calcification (*p* < .0005; Figure 4). There was no relation between the occurrence of medial calcification and intimal calcification. Medial calcification was present in 39/75 samples (52%) with intimal calcification and in 106/195 samples (54%) without intimal calcification (*p* = .728).

## 4 DISCUSSION

We studied the natural development of atherosclerosis and vascular wall calcification in the leg of a general elderly population. The study has three important results. First, atherosclerotic lesions were more often found in samples of the popliteal artery than in samples of the more distally located posterior tibial artery, but this did not result in a higher percentage stenosis in the popliteal artery. Second, in both artery types atherosclerotic lesions were most often non‐atheromatous fibrotic lesions, although also atheromatous (thin) fibrous cap atheroma's were observed in both artery types. Third, atherosclerotic intimal calcification was more frequently present in samples of the popliteal artery than in samples of the posterior tibial artery, whereas medial arterial calcification was more often present in samples of the posterior tibial artery than in samples of the popliteal artery.

In the below the knee arteries of the leg we observed mostly intimal thickening and fibrous atherosclerotic lesions and only in a minority of arteries atheromatous lesions. This observation is in concordance with observations in amputation specimens of patients with peripheral artery disease (PAD) in which also predominantly nonatheromatous fibrotic lesions have been described, whereas atheromatous atherosclerotic lesions were found in a relatively low frequency (O'Neill, Han, Schneider, & Hennigar, 2015; Soor, Vukin, Leong, Oreopoulos, & Butany, 2008). Furthermore, in a recent study in deceased patients with abundant cardiovascular risk factors, below‐the‐knee lesions were also more often of the fibrous type (Torii et al., 2019). In this previous patient study, 10 below the knee arteries revealed total occlusions, of which half were embolic in origin and half were due chronic total occlusion. In contrast to the below the knee arteries, in this study all 8 arterial occlusions above the knee were associated with acute thrombus formation, of which 3 were associated with plaque rupture of an atheromatous plaque and 5 were related to calcified nodules (Torii et al., 2019). We did not observe emboli and in one leg we observed a total occlusion in the posterior tibial artery due to a ruptured fibrous cap atheroma. In our study even in the atheromatous lesions almost all atheroma's showed a lipid pool that was relatively small (<40% of the intimal surface). Our findings support the hypothesis that also in the general elderly population, arteries below the knee are less prone for the development of atheromatous lesions with an extracellular lipid core.

We observed medial arterial calcification more often in samples of the posterior tibial artery (62%) than in samples of the popliteal artery (46%), whereas atherosclerotic intimal calcification was more frequently present in samples of the popliteal artery (39%) than in the posterior tibial artery (17%). Although medial calcification is frequently observed in amputation specimens, the clinical significance remains unclear (O'Neill et al., 2015; Soor et al., 2008). We did not find a correlation between medial arterial calcification and the amount of stenosis, which is in concordance with the results of a study in amputation specimens (O'Neill et al., 2015). However, in some patients medial arterial calcification has been suggested to be the cause of amputation (Soor et al., 2008). Arterial medial calcification is thought to be associated with stiffening of the vascular wall (Kamenskiy et al., 2018), which could lead to an increase in pulse wave velocity, an increase in pulse pressure and pulse wave deformation (Lanzer et al., 2014). Furthermore, arterial medial calcification interferes with positive arterial remodeling, which could lead to more severe morbidity due to coincident intimal lesions (Lanzer et al., 2014). Previous studies in breast arterial calcifications (which are thought to be exclusively medial [Duhn et al., 2011]) and genetic types of medial arterial calcification have shown regression of calcifications, either spontaneous or with treatment (Edouard et al., 2011; Hendriks et al., 2015; Kranenburg et al., 2018). The finding of both intimal and medial arterial calcification in the arteries of the lower extremity suggests the need for a reliable in vivo method to discriminate between both types of calcification. This would enable further study into the clinical significance and potential treatment of medial arterial calcifications.

An important limitation of this study is the use of a relatively small number of anonymously donated specimens of which medical history and cardiovascular risk factors are unknown.

In conclusion, in the leg of an elderly population, the presence of atherosclerotic plaque and intimal calcification decreases from the proximal popliteal artery to the more distal posterior tibial artery and most atherosclerotic lesions are of the nonatheromatous type. In contrast, the presence and severity of medial calcification increases from proximal to distal.

## CONFLICT OF INTEREST

The authors have no conflict of interest.
